# Supplementary material for: Accelerated absorption of regular insulin administered via a vascularizing permeable microchamber implanted subcutaneously in diabetic Rattus norvegicus
Source: PLoS One. 2023 Jun 29;18(6):e0278794. doi: 10.1371/journal.pone.0278794 (PMC10310011; doi:10.1371/journal.pone.0278794)
Supplement: Attachment — Submitted filename: Review comments.docx [file pone.0278794.s001.docx]

The manuscript submitted on the titled, "Accelerated absorption of regular insulin administered via a vascularizing permeable microchamber implanted subcutaneously in diabetic Rattus norvegicus" is worth publishing in the PLOS ONE journal, with further minor revision of the manuscript.

1. What are the triggers to induce vascularization in the inserted microchamber?

2. Have you observed any allergic reactions after implanting the microchamber?
