# Supplementary material for: Accelerated absorption of regular insulin administered via a vascularizing permeable microchamber implanted subcutaneously in diabetic Rattus norvegicus
Source: PLoS One. 2023 Jun 29;18(6):e0278794. doi: 10.1371/journal.pone.0278794 (PMC10310011; doi:10.1371/journal.pone.0278794)
Supplement: Attachment — Submitted filename: Response to Reviewers-FINAL.docx [file pone.0278794.s002.docx]

P. Balaji, Ph.D.

Academic Editor

PLOS ONE

Dear Dr. Balaji,

We would like to thank you, as well as your staff and the two Reviewers, for your careful and thoughtful review of our manuscript. We appreciate the time commitment and the helpful suggestions which have improved the quality of our submission. Below, we first address the Journal Requirements, then the concerns and questions posed by the two Reviewers.

**Journal Requirements:**

1) In the Methods section, we have added detailed information regarding the animal experiments, including specific details regarding *methods of sacrifice*, *methods of anesthesia and analgesia*, and *efforts to alleviate suffering* (Please see the “Revised Manuscript with Track Changes”).

2) We wish to amend the Competing Interests section, in conformance with the journal’s policy:

“I have read the journal's policy and the authors of this manuscript have the following competing interests: [R.J. and KKP are co-founders of and have financial interests in Procyon Technologies LLC.]. *This does not alter our adherence to PLOS ONE policies on sharing data and materials.”*

We have responded by email (April 16, 2023), indicating our desire to have our Competing Interests statement amended.

3) We have included the full Ethics statement in the Methods section of our revised manuscript (see “Revised Manuscript with Track Changes”). It reads:

**Ethical care of experimental animals**

All experiments were performed with the written approval of and in accordance with the guidelines established by the Institutional Animal Care and Use Committee at The University of Arizona.

4) We have carefully reviewed our reference list for accuracy and have diligently checked it for Retractions. As of April 15, 2023, none of our cited references had been flagged as “retraction” or “retracted”. We have added three references in response to queries by the Reviewers. These references are listed below:

43.	Geller RL, Loudovaris T, Neuenfeldt S, Johnson RC, Brauker JH. Use of an immunoisolation device for cell transplantation and tumor immunotherapy. Ann N Y Acad Sci. 1997;831:438-51. doi: 10.1111/j.1749-6632.1997.tb52216.x. PubMed PMID: 9616733.

44.	Goyal SN, Reddy NM, Patil KR, Nakhate KT, Ojha S, Patil CR, et al. Challenges and issues with streptozotocin-induced diabetes - A clinically relevant animal model to understand the diabetes pathogenesis and evaluate therapeutics. Chem Biol Interact. 2016;244:49-63. Epub 20151202. doi: 10.1016/j.cbi.2015.11.032. PubMed PMID: 26656244.

45.	Bartholomaeus WN, O'Donoghue H, Reed WD. Thymus-dependence of autoantibody responses to liver specific lipoprotein in the mouse. Clin Exp Immunol. 1984;55(3):541-5. PubMed PMID: 6323073; PubMed Central PMCID: PMCPMC1535937.

**Reviewers’ Comments**

**Reviewer #1:**

1. What was the reason behind selecting the immunodeficient rat for the study?

Because the rats were to receive human insulin, we were concerned that a potential antibody response to the human protein by the subject rat might sequester or otherwise interfere with measurements of the insulin kinetic response. The nude rodent model is often used to avoid interference by the immune system in xenogeneic systems (most commonly, in human tumor models). However, the athymic nude model has also been used to avert immune antibody responses to xeno-proteins [1]. (We have added this citation to the manuscript.)

2. Why particular sex(male) being preferred for the study?

Thank you for raising this question! Even investigators working with the streptozotocin (STZ)-induced diabetes model may not be aware that estrogen interferes with the action of STZ in inducing diabetes; hence males are more sensitive to STZ and can be induced with lower doses, thereby diminishing off-target toxicities [2]. (We have also added this citation to the manuscript.)

3. Kindly specify the significant contributing factor (Increasing vascular permeability or novelty of pumping design or location of implantation or if any other factors) for rapid insulin absorption in the conclusion.

Your suggestion is an excellent one and we have revised the **Conclusion** to clearly state that our data indicate that robust neovascularization abetted by the outer membrane of the IVM device is important to rapid insulin absorption; the **Conclusion** now reads in part: “…neovascularization surrounding the IVMs was a significant contributor to the rapidity of insulin uptake.”

**Reviewer # 2:**

1. What are the triggers to induce vascularization in the inserted microchamber?

Membrane microarchitecture [3, 4] is the established trigger of neovascularization surrounding our microchambers. The outer (vascularizing) membrane of the mirochamber is composed of a PTFE (i.e. Teflon), having pores of ~5µm. These pores enable ingrowth of smaller blood vessels facilitating neovascularization, which is 80 – 100X that found surrounding a 0.02 µm PTFE membrane that does not allow cells to penetrate it [3]. (Both citations are now included in the manuscript). We have revised the opening paragraph of the **Discussion** to emphasize these points (see Manuscript with Track Changes).

2. Have you observed any allergic reactions after implanting the microchamber?

The devices were fashioned of PTFE in large part because of its many-years’ record of successful use in humans [5]. In fact, it is so hypo-allergenic that it has been used to wrap metal or silastic devices in patients having allergic responses to the latter materials. We did not observe any signs or symptoms of systemic or local allergic reactions to the microchambers in the rats; moreover, the histology of the devices after removal showed well-developed vascularization and integration with the host tissues.

In addition to this “Response to the Reviewers”, we have uploaded the “Revised Manuscript with Track Changes” and the unmarked version, “Manuscript.” We appreciate your careful consideration of our responses and look forward to our report appearing in PLOS ONE!

Sincerely,

Charles W. Putnam	Klearchos K. Papas

Submitting Author	Corresponding Author

References cited in response to Reviewers’ queries:

1.	Bartholomaeus WN, O'Donoghue H, Reed WD. Thymus-dependence of autoantibody responses to liver specific lipoprotein in the mouse. Clin Exp Immunol. 1984;55(3):541-5. PubMed PMID: 6323073; PubMed Central PMCID: PMCPMC1535937.

2.	Goyal SN, Reddy NM, Patil KR, Nakhate KT, Ojha S, Patil CR, et al. Challenges and issues with streptozotocin-induced diabetes - A clinically relevant animal model to understand the diabetes pathogenesis and evaluate therapeutics. Chem Biol Interact. 2016;244:49-63. Epub 20151202. doi: 10.1016/j.cbi.2015.11.032. PubMed PMID: 26656244.

3.	Brauker JH, Carr-Brendel VE, Martinson LA, Crudele J, Johnston WD, Johnson RC. Neovascularization of synthetic membranes directed by membrane microarchitecture. J Biomed Mater Res. 1995;29(12):1517-24. doi: 10.1002/jbm.820291208. PubMed PMID: 8600142.

4.	Geller RL, Loudovaris T, Neuenfeldt S, Johnson RC, Brauker JH. Use of an immunoisolation device for cell transplantation and tumor immunotherapy. Ann N Y Acad Sci. 1997;831:438-51. doi: 10.1111/j.1749-6632.1997.tb52216.x. PubMed PMID: 9616733.

5.	Yorita K, Takahashi J, Tano K, Ichikawa Y, Hamaguchi N, Yasui W. Twenty years' use of expanded polytetrafluoroethylene sheet for an artificial cardiac pacemaker. Clin Case Rep. 2021;9(6):e04334. Epub 20210624. doi: 10.1002/ccr3.4334. PubMed PMID: 34194807; PubMed Central PMCID: PMCPMC8223883.
